# Asthma and Chronic Obstructive Pulmonary Disease (COPD) Prevalence and Health Services Use in Ontario Métis: A Population-Based Cohort Study

**DOI:** 10.1371/journal.pone.0095899

**Published:** 2014-04-23

**Authors:** Andrea S. Gershon, Saba Khan, Julie Klein-Geltink, Drew Wilton, Teresa To, Eric J. Crighton, Lisa Pigeau, Jo MacQuarrie, Yvon Allard, Storm J. Russell, David A. Henry

**Affiliations:** 1 Department of Medicine, Sunnybrook Health Sciences Centre, Toronto, Ontario, Canada; 2 Institute for Clinical Evaluative Sciences, Toronto, Ontario, Canada; 3 Child Health Evaluative Sciences, The Hospital for Sick Children, Toronto, Ontario, Canada; 4 University of Toronto, Toronto, Ontario, Canada; 5 Department of Geography, University of Ottawa, Ottawa, Ontario, Canada; 6 Métis Nation of Ontario, Ontario, Canada; New York University School of Medicine, United States of America

## Abstract

**Introduction:**

Chronic respiratory diseases cause a significant health and economic burden around the world. In Canada, Aboriginal populations are at increased risk of asthma and chronic obstructive pulmonary disease (COPD). There is little known, however, about these diseases in the Canadian Métis population, who have mixed Aboriginal and European ancestry. A population-based study was conducted to quantify asthma and COPD prevalence and health services use in the Métis population of Ontario, Canada’s largest province.

**Methods:**

The Métis Nation of Ontario Citizenship Registry was linked to provincial health administrative databases to measure and compare burden of asthma and COPD between the Métis and non-Métis populations of Ontario between 2009 and 2012. Asthma and COPD prevalence, health services use (general physician and specialist visits, emergency department visits, hospitalizations), and mortality were measured.

**Results:**

Prevalences of asthma and COPD were 30% and 70% higher, respectively, in the Métis compared to the general Ontario population (p<0.001). General physician and specialist visits were significantly lower in Métis with asthma, while general physician visits for COPD were significantly higher. Emergency department visits and hospitalizations were generally higher for Métis compared to non-Métis with either disease. All-cause mortality in Métis with COPD was 1.3 times higher compared to non-Métis with COPD (p = 0.01).

**Conclusion:**

There is a high burden of asthma and COPD in Ontario Métis, with significant prevalence and acute health services use related to these diseases. Lower rates of physician visits suggest barriers in access to primary care services.

## Introduction

Chronic respiratory diseases, including asthma and chronic obstructive pulmonary disease (COPD), are a leading cause of morbidity and mortality worldwide [1]. Globally, 235 million people are believed to suffer from asthma, while COPD is the 4^th^ leading cause of death [2], [3]. In Canada, asthma affects about 13% of the population and is the most common chronic disease of childhood [4]. COPD (also known as emphysema or chronic bronchitis) affects about 10% of the Canadian adult population and is a leading cause of chronic disease hospitalization [5], [6]. Altogether, chronic respiratory diseases are estimated to cost $154 billion per year in direct and indirect costs nationally [7].

Aboriginal peoples have been found to be at increased risk of chronic respiratory diseases in many countries, including Canada [8]. In a recent Canadian survey study, self-reported prevalence of respiratory disease was significantly higher in Aboriginal peoples aged 15 and older compared to non-Aboriginals (15% vs. 10%) [9]. Aboriginal peoples in Canada also appear to have higher rates of respiratory disease-related emergency department visits and mortality [10], [11]. However, most respiratory disease studies in the Canadian Aboriginal population have focused on First Nations and Inuit peoples; relatively little is known about these diseases in the Métis population.

The Métis are one of the founding Aboriginal Peoples of Canada, descended from unions between First Nations women and European men. In the 2011 Canadian National Household Survey, 451,795 people self-identified as Métis, accounting for 32% of the overall Aboriginal population in Canada [12]. The Métis are a federally recognized and distinct Aboriginal people with unique language, culture, and history. Like First Nations and Inuit peoples, the Métis suffer a disproportionate burden of ill health compared to the rest of the Canadian population. In the 2006 Canadian Aboriginal People’s Survey (APS), more than 50% of Métis reported being diagnosed with a chronic condition and almost one-third reported having two or more chronic conditions [13]. However, unlike First Nations and Inuit, the Métis do not receive federal benefits, such as post-secondary tuition support, income assistance or comprehensive prescription drug coverage. They also receive significantly fewer local, provincial and national health care resources and rarely have their own dedicated health centres [14].

Studies based on the 2001 and 2006 cycles of the APS suggest that asthma is more prevalent among the Métis than other adults in Canada [13], [15]. However, asthma estimates in these studies were based on self-report and therefore subject to recall bias. Two previous studies have used health administrative data to demonstrate significantly higher overall respiratory morbidity and mortality in the Métis, but did not fully sub-analyze results by disease type [11], [16]. We are not aware of any previous literature quantifying the burden of COPD in Métis populations in particular. Thus, there is still relatively little known about the burden of asthma and COPD among Métis peoples in Canada, and no such information specific to Ontario, Canada’s most populous province, where Métis people account for approximately 30% of the total Aboriginal population [12].

To address this research gap, we used health administrative data to conduct a population-based study to quantify asthma and COPD burden in the Ontario Métis population, in terms of prevalence, health services use, and outcomes. To contextualize the results, we compared asthma and COPD in the Métis to the rest of the Ontario population.

## Methods

### Ethics Statement

The research described here was commissioned by the Métis Nation of Ontario (MNO) and conducted at the Institute for Clinical Evaluative Sciences (ICES). This study was approved by the Research Ethics Board of Sunnybrook Health Sciences Centre, Toronto, Ontario and was conducted under guidelines for research with Aboriginal communities that have been developed by Canada’s three principal research agencies [17], as well as the MNO’s internal guidelines for collaborative research with external partners. This study used routinely collected, anonymized health information from the province of Ontario and did not require informed consent from participants. ICES is named as a prescribed entity under section 45 of the *Personal Health Information Protection Act* (Ontario Regulation 329/04, Section 18). Under this designation, ICES can receive and use personal health information in a de-identified manner without consent for purposes of analysis and compiling statistical information about the health care system of Ontario.

### Study Design and Setting

We conducted a population-based study from April 1, 2009 to March 31, 2012 by linking the Métis Nation of Ontario Citizenship Registry to health administrative data from Ontario [18], the largest province of Canada with a population of approximately 13.5 million.

### Data Sources

Our source population was identified in the Métis Nation of Ontario (MNO) Citizenship Registry, which contains information on all Métis citizens who are able to supply genealogical documentation and historical proof of Métis ancestry. Probabilistic linkage of this database to the health administrative data on an individual level was performed with 97% success. Residents of Ontario are insured under the Ontario Health Insurance Plan (OHIP). Personal health information is captured in provincial health administrative databases which can be linked together, using unique health insurance numbers, to give complete health services use profiles of all those living in the province. In this study, six health administrative databases were used. The Registered Persons Database is a central registry of all insured persons in Ontario which also records death information, where appropriate. The Ontario Health Insurance Program claims database contains all fee-for-service billing claims from physicians for consultations, visits and procedures. The National Ambulatory Care Reporting System contains diagnostic information related to all emergency department visits. The Discharge Abstracts Database contains information about all hospitalizations including diagnoses and procedures performed.

Finally, the Ontario Asthma Surveillance Information System and the Ontario Chronic Obstructive Pulmonary Disease Databases are validated databases of all people in Ontario with asthma and COPD [19], [20]. Both were developed using validated case definitions with the health administrative databases described above (Table 1). Of note, Ontario health administrative databases also contain information on prescription medication treatment, but only for people aged 65 and over or on social assistance. As less than 10% of the Métis population in this study were aged 65 and older, we did not have a suitable population size to examine prescription medication use, a mainstay of the treatment of COPD and asthma [1].

**Table 1:** Health administrative data case definitions for asthma and chronic obstructive pulmonary disease (COPD).

Disease	Health AdministrativeData Case Definition	International Classificationof Diseases-9 andOntario Health InsuranceProgram Codes forhospitalizations priorto 2002 andambulatory care visits	International Classificationof Diseases-10 Codesfor hospitalizationsafter 2002	Sensitivity	Specificity
Asthma^1^	1 asthma hospitalizationand/or 2 asthma ambulatorycare visits within 2 years	493	J45, J46	84%	76%
Chronicobstructivepulmonarydisease^2^	1 COPD hospitalizationand/or 1 COPD ambulatory inan adult aged 35 and older	491, 492, 496	J41, J43, J44	85%	78%

1 Gershon AS, Wang C, Vasilevska-Ristovska J, Guan J, Cicutto L, et al. (2009) Identifying patients with physician diagnosed asthma in health administrative databases. Can Respir J 16∶183–188.

2 Gershon AS, Wang C, Guan J, Vasilevska-Ristovska J, Cicutto L, et al. (2009) Identifying individuals with physician diagnosed COPD in health administrative databases. COPD 6∶388–394.

### Study Population

All individuals recorded in the Métis registry and successfully linked with health administrative data were included and hereafter are referred to as “the Métis”. All other Ontario residents were considered the general population. Both populations were further limited to people who were alive, living in Ontario and eligible for health insurance as of April 1, 2009. When considering asthma outcomes, we excluded individuals under the age of 20 as the Métis Registry underrepresents this age group. When considering COPD outcomes, we excluded individuals under the age of 35 as COPD is rare in younger individuals.

### Outcomes

People with prevalent asthma and/or COPD were identified as of April 1, 2009 using the validated asthma and COPD databases described above. They were then followed for three years to determine if they died. Those who were still alive had their health services utilization measured during the follow-up period. We looked at disease-specific and all-cause ambulatory care visits (primary care practitioner and specialist visits), emergency department visits, and hospitalizations.

### Covariables

To account for differences between the Métis and the rest of the Ontario population, demographics such as age, sex, and geographic variables were obtained using the Registered Persons Database. Geographic variables, generated using the Statistics Canada Postal Code Conversion File [21], included geographic region, neighbourhood income quintile, and urban/rural status (see Table 2 footnotes for definitions).

**Table 2:** Characteristics of the Métis and the general Ontario study populations, as of April 1, 2009^1^.

Characteristic	Métis	Ontario
**Number**	12,350	9,833,152
**Mean age (SD)**	45.9 (15.1)	47.8 (17.3)
**Age groups (%)**		
20–39	36.4	35.4
40–59	44.3	39.5
60 and older	19.3	25.1
**Sex (%)**		
Male	53.3	48.4
Female	46.7	51.6
**Geographic region (%)**		
Southern Ontario	53.3	93.6
Northern Ontario	46.7	6.4
**Neighbourhood income quintile (%)** 2		
1 (poorest/lowest)	22.1	19.3
2	20.9	19.9
3	20.3	19.9
4	18.8	20.5
5 (richest/highest)	17.3	20.1
**Urban/rural status (%)** 3		
Urban	69.3	88.3
Rural	30.7	11.7

1 Métis and general population cohorts were limited to persons aged 20+, alive, living in Ontario and eligible for health insurance.

2 Neighbourhood income was calculated by Statistics Canada. Canadian neighbourhoods are classified into one of five approximately equal-sized groups (quintiles), ranked from poorest (quintile1) to wealthiest (quintile 5).

3 Based on the Statistics Canada definition of rurality, where Census Metropolitan Areas that have a population less than 10,000 are considered to be rural.

### Statistical Analysis

Counts and proportions were generated for all covariables in the Métis and Ontario populations. Asthma and COPD prevalence were generated overall and by age group, sex and urban/rural status for both the Métis and the general Ontario population. Chi-square tests were used to test for differences, with p-values less than 0.05 considered significant. Rates of ambulatory care visits, emergency department visits, hospitalizations and mortality were calculated per 1000 person-years. Due to the differences in demographic structure and geographic location of the two study populations, all rates were direct-standardized for age, sex, and urban/rural status to the 2006 Ontario Census population. 95% confidence intervals were calculated using the gamma method [22].

## Results

A total of 12,350 Métis were included in this study. Compared to the general population cohort (N = 9,833,152), the Métis were slightly younger and a higher percentage were male and resided in Northern, rural, and lower-income areas of the province (Table 2).

### Prevalence

In the Métis, the overall crude prevalence of asthma was 15% (Table 3). Prevalence was highest in the younger age groups, females, and those living in urban areas. The standardized asthma prevalence was 1.3 times higher in the Métis than the general Ontario population (16% vs. 12%, p<0.001).

**Table 3:** Prevalence of asthma and chronic obstructive pulmonary disease (COPD) in the Métis and Ontario general populations, as of April 1, 2009.

	ASTHMA			CHRONIC OBSTRUCTIVEPULMONARY DISEASE		
	Métis	Ontario	P-value	Métis	Ontario	P-value
**Number**	1797	1,170,139		1309	696,242	
**Crude rate** 1	14.6 (13.9–15.2)	11.9 (11.9–11.9)	<0.001	16.7 (15.8–17.6)	11.0 (10.9–11.0)	<0.001
**Standardized rate** 2	15.6 (14.8–16.4)	11.8 (11.8–11.8)	<0.001	17.3 (16.1–18.5)	10.2 (10.1–10.2)	<0.001
**Age group (%)**						
20–29	23.7	24.1		N/A	N/A	
30–39	20.1	16.5		N/A	N/A	
40–49	21.3	18.6		15.8	12.1	
50–59	17.5	16.0		29.6	22.8	
60–69	11.7	11.5		30.1	24.2	
70–79	4.3	8.0		18.3	22.7	
80 and older	1.5	5.2		6.2	18.2	
**Sex (%)**						
Male	42.3	41.0		53.0	48.9	
Female	57.7	59.0		47.0	51.1	
**Urban/rural status (%)**						
Urban	74.2	89.0		66.2	83.6	
Rural	25.8	11.0		33.8	16.4	

N/A: Not applicable.

1 Crude rate per 100 people (95% confidence interval).

2 Direct-standardized rate by age, sex and urban/rural status to the 2006 Ontario Census population.

Crude COPD prevalence was 17% in the Métis and was highest in the 50 to 69 year age group, males, and urban dwellers. The standardized COPD prevalence was 1.7 times higher in the Métis than in the general Ontario population (17% vs. 10%, p<0.001).

### Health Services Use

Between 2009 and 2012, more than 90% of both Métis and non-Métis people in the province with asthma or COPD visited a primary care practitioner (Table 4). Rates of all-cause and asthma-specific primary care practitioner visits were significantly lower in Métis with asthma compared to other Ontarians with asthma. Likewise, rates of specialist visits were also lower, especially asthma-specific visits, which were less than half the rate of the general population with asthma. During the same time, rates of all-cause and COPD-specific primary care practitioner visits in Métis with COPD were higher or comparable to rates in non-Métis with COPD. Rates of specialist visits, however, were lower, especially rates of COPD-specific visits, which were less than one third the rate of the general population.

**Table 4:** Ambulatory care visits by people with asthma and chronic obstructive pulmonary disease (COPD) in the Métis and Ontario general populations, April 1, 2009 to March 31, 2012^1^.

	ASTHMA			CHRONIC OBSTRUCTIVEPULMONARY DISEASE		
	Métis	Ontario	P-value	Métis	Ontario	P-value
**All-cause primary care** **practitioner visits**						
N (%) with visit	613 (91.4)	1,058,482 (92.5)		1160 (94.5)	603,657 (94.7)	
Standardized rate	4984.6(4896.2–5074.1)	5374.5(5371.3–5377.6)	<0.001	6171.5(6042.3–6302.7)	6031.3(6025.9–6036.7)	0.005
**Disease-specific primary care** **practitioner visits**						
N (%) with visit	319 (18.1)	237,280 (20.7)		243 (19.8)	105,014 (16.5)	
Standardized rate	178.2(162.7–194.7)	224.8(224.2–225.5)	<0.001	201.8(181.2–224.1)	175.0(174.2–175.8)	0.002
**All-cause specialist visits**						
N (%) with visit	1143 (64.8)	753,899 (65.9)		923 (75.2)	495,581 (77.7)	
Standardized rate	2334.2(2273.6–2395.9)	2433.4(2431.2–2435.5)	<0.001	2831.8(2746.2–2919.5)	2931.5(2927.9–2935.1)	0.004
**Disease-specific specialist** **visits**						
N (%) with visit	60 (3.4)	65,880 (5.8)		37 (3.0)	40,491 (6.4)	
Standardized rate	32.4 (26.4–29.4)	69.3(68.9–69.7)	<0.001	20.7 (15.2–27.5)	64.6(64.1–65.1)	<0.001

1 All rates per 1000 person-years, direct-standardized to the 2006 Ontario Census population by age, sex and urban/rural status (with 95% confidence intervals).

During the study period, about 60% of Métis with either asthma or COPD visited an emergency department, although only between 5 and 6% had an asthma- or COPD-specific visit (Table 5). Sixteen and 24% of Métis with asthma and COPD, respectively, were hospitalized for any cause. After standardization, rates of all-cause and disease-specific emergency department visits and hospitalizations in the Métis with asthma or COPD were generally higher than the rest of the Ontario population, with the exception of COPD-specific ED visits, which were slightly lower in the Métis.

**Table 5:** Emergency department visits and hospitalizations by people with asthma and chronic obstructive pulmonary disease (COPD) in the Métis and Ontario general populations, April 1, 2009 to March 31, 2012^1^.

	ASTHMA			CHRONIC OBSTRUCTIVE PULMONARY DISEASE		
	Métis	Ontario	P-value	Métis	Ontario	P-value
**All-cause emergency department visits**						
N (%) with visit	1031 (58.5)	504,900 (44.1)		731 (59.6)	319,222 (50.1)	
Standardized rate	761.7 (729.0–795.8)	563.9 (562.9–565.0)	<0.001	910.7 (861.3–962.2)	674.3 (672.5–676.2)	<0.001
**Disease-specific emergency department visits**						
N (%) with visit	85 (4.8)	33,768 (3.0)		75 (6.1)	32,266 (5.1)	
Standardized rate	26.3 (21.2–32.3)	21.5 (21.3–21.7)	0.05	26.7 (20.4–34.3)	32.2 (31.9–32.5)	0.13
**All-cause hospitalizations**						
N (%) with visit	280 (15.9)	152,486 (13.3)		293 (23.9)	136,821 (21.5)	
Standardized rate	118.9 (104.5–134.7)	93.1 (92.6–93.5)	<0.001	176.3 (155.9–198.5)	132.7 (132.0–133.4)	<0.001
**Disease-specific hospitalizations**						
N (%) with visit	20 (1.1)	9903 (0.9)	0.31	66 (5.4)	32,823 (5.2)	
Standardized rate	5.8 (3.2–9.8)	4.7 (4.6–4.8)		32.9 (25.1–42.3)	25.0 (24.7–25.3)*	0.01

1 All rates per 1000 person-years, direct-standardized to the 2006 Ontario Census population by age, sex and urban/rural status (with 95% confidence intervals).

### Mortality

Standardized all-cause mortality was 1.3 times higher for Métis compared to other Ontarians with COPD (39.0 vs. 29.0 deaths per 1000 people, p = 0.01). Standardized all-cause mortality was similar between Métis and non-Métis with asthma (68.0 vs. 65.0 deaths per 1000 people, p = 0.67).

## Discussion

We conducted a population-based cohort study of the Ontario population that confirmed that Métis people living in Ontario had a higher prevalence of physician-diagnosed asthma and COPD than the rest of the Ontario population. We also found generally lower rates of primary care practitioner and specialist visits for these diseases among the Métis, suggesting disparity in access to and/or utilization of ambulatory health care for this group of Ontarians. These results could be contributing the higher rates of emergency department visits and hospitalizations also observed for asthma and COPD among Métis populations.

To the best of our knowledge, this is the first large-scale, population study to quantify asthma and COPD in the Ontario Métis population in terms of health services use. Its results point to a disturbing gap in respiratory health between the Métis and non-Métis that requires further study.

Our findings are consistent with the self-reported asthma prevalence rates of the Métis who participated in the 2006 Aboriginal People’s Survey [13]. Our results also concur with a Manitoba study that found total respiratory morbidity (a mixed measure of asthma, bronchitis, emphysema and chronic airway obstruction burden) to be significantly higher in the Manitoba Métis compared to the general population [16].

A number of factors are likely contributing to the higher prevalence of asthma and COPD observed among the Ontario Métis. The prevalence of smoking, a known risk factor for COPD and a trigger for asthma, is 33% for the Métis population nationally, nearly twice that of the Canadian general population [23]. Socio-economic conditions, such as poor housing with poor air quality and enhanced exposure to allergens and molds, may also be contributors in regions where many Métis reside [24].

We also found disparities in health services use, with generally lower rates of ambulatory care visits observed among the Métis, along with higher rates of acute care services. These results are similar to those found in an Alberta study, where specialist visit rates for asthma and COPD were lower in Aboriginal peoples than the general population, despite significantly higher disease rates [25]. The exception in our study was a slightly higher rate of all-cause and COPD-specific primary care practitioner visits among Métis people with COPD, which could be indicative of more severe disease or more comorbidity in this population. Geographical factors may also be a contributing factor in the higher rates of acute care service usage. Many Métis people live in smaller and more remote communities, where timely access to specialist Métis care and diagnostics is a challenge. Without this access, many Métis with asthma and COPD may be ending up in the emergency department or hospital for care [24]. This is especially a cause for concern for Métis with COPD, as their mortality rates were higher than other Ontarians with COPD.

The strengths of our study were its population base, comprehensiveness and ability to capture and compare rates in the Métis to a general non-Métis population. It also has limitations which merit emphasis. First, while use of health administrative data has been widely advocated for use in chronic disease surveillance as an efficient, available and relatively inexpensive way to obtain population-based measures of disease burden [26], [27], such data also have limitations including possible misclassification of disease and lack of clinical information, such as information on pulmonary function testing and smoking status. Nonetheless, we used validated case definitions of asthma and COPD with good sensitivity and specificity [19], [20].

A second important limitation is that the Citizenship Registry of the MNO is voluntary and does not include all Métis in the province; as such, it may not be representative of the entire Métis population. However, it is the largest, most complete registry of Métis in Ontario and provides the best representation of Métis respiratory health currently available. Our results are also likely a conservative estimate of the differences between Métis and non- Métis, since any non-registered Métis people would have been included in the general population of this study, thereby minimizing any true differences between Métis and non-Métis populations. Furthermore, Métis citizens, by virtue of the fact that they had the capacity, energy and motivation to undertake the comprehensive process required to register and be recognized as citizens, are likely healthier than non-registered Métis residents, making our results even more likely to be conservative estimates of disease in this population.

Finally, the Métis registry has few people under the age of 18 and so we were unable to look at the burden of asthma in children. As asthma tends to have a higher burden among the younger age groups, this may be another reason why our results underestimate the total burden of asthma in the Métis. The MNO is currently working towards increasing the registration of children in their Citizenship Registry, and this additional data will be valuable in examining this issue.

In summary, we conducted a population-based cohort study and found that citizens of the Métis Nation of Ontario had higher rates of asthma and COPD and related acute health services use than the general Ontario population. These analyses suggest deficiencies in primary health care access for these diseases for the Métis that make this population more reliant on emergency services to address their health care needs. Future research should focus on confirming these findings in the entire Métis population, examining respiratory conditions in younger populations, identifying specific factors that are contributing to higher rates of asthma and COPD among the Métis compared to the rest of the Ontario population, and determining strategies to minimize the disease burden for this at-risk population.
